# Elevation is Associated with Human Skin Microbiomes

**DOI:** 10.3390/microorganisms7120611

**Published:** 2019-11-24

**Authors:** Huan Li, Yijie Wang, Qiaoling Yu, Tianshu Feng, Rui Zhou, Liye Shao, Jiapeng Qu, Nan Li, Tingbei Bo, Huakun Zhou

**Affiliations:** 1 School of Public Health, Lanzhou University, Lanzhou 730000, China; 2 Key Laboratory of Restoration Ecology of Cold Area in Qinghai Province, Northwest Institute of Plateau Biology Chinese Academy of Sciences, Xining 810008, China; 3 Key Laboratory of Health Aquaculture and Product Processing in Dongting Lake Area of Hunan Province, Zoology Key Laboratory of Hunan Higher Education, Hunan University of Arts and Science, Hunaan Changde 415000, China; 4 Key Laboratory of Adaptation and Evolution of Plateau Biota, Northwest Institute of Plateau Biology, Chinese Academy of Sciences, Xining, Qinghai 810008, China; 5 Key Laboratory of Environment Change and Resources Use in Beibu Gulf, Ministry of Education, Nanning Normal University, 175 Mingxiu East Road, Nanning, Guangxi 530001, China; 6 State Key Laboratory of Integrated Management of Pest Insects and Rodents, Institute of Zoology, Chinese Academy of Sciences, Beijing 100101, China

**Keywords:** Skin microbiome, elevation, diversity, network, deterministic processe

## Abstract

Human skin microbiota plays a crucial role in the defense against pathogens, and is associated with various skin diseases. High elevation is positively correlated with various extreme environmental conditions (i.e., high ultraviolet radiation), which may exert selection pressure on skin microbiota, and therefore influence human health. Most studies regarding skin microbial communities have focused on low-elevation hosts. Few studies have explored skin microbiota in high-elevation humans. Here, we investigated the diversity, function, assembly, and co-occurrence patterns of skin microbiotas from 35 health human subjects across three body sites (forehead, opisthenar, and palm) and seven elevation gradients from 501 to 3431 m. Alpha diversity values (i.e., Shannon diversity and observed operational taxonomic units (OTUs)) decreased with increasing elevation regardless of the body site, while beta diversity (Jaccard and Bray–Curtis dissimilarities) showed an increasing trend with elevation. Elevation is a significant factor that influences human skin microbiota, even after controlling host-related factors. Skin microbiotas at high elevation with more than 3000 m on the Qinghai–Tibet Plateau, had a significant structural or functional separation from those at low elevation with less than 3000 m. Notably, the clustering coefficient, average degree, and network density were all lower at high-elevation than those at low-elevation, suggesting that high-elevation skin networks were more fragile and less connected. Phylogenetic analysis showed that human skin microbiotas are mainly dominated by stochastic processes (58.4%–74.6%), but skin microbiotas at high-elevation harbor a greater portion of deterministic processes than those at low-elevation, indicating that high-elevation may be conducive to the promotion of deterministic processes. Our results reveal that the filtering and selection of the changeable high-elevation environment on the Qinghai–Tibet Plateau may lead to less stable skin microbial community structures.

## 1. Introduction

Skin can be regarded as the largest human organ, and also as the first important line of defense against external pathogens and toxic substances by secreting antimicrobial peptides, salts, enzymes, lipids, and many other compounds [1]. Simultaneously, human skin harbors diverse microbial communities, including bacteria, archaea, fungi, and viruses [2,3]. These symbiotic microorganisms play a crucial role in host physiology, such as improving colonization resistance to transient microbes, impacting lipid metabolism, and educating immunity [1,4,5]. In addition, skin microbiota composition are also impacted by various environmental and host factors, such as pH, moisture, temperature, host age, sex, body site, and species [6,7,8,9,10,11]. Some reports have demonstrated a pivotal function of skin microbial communities in regulating the health and environmental adaptability of humans and animals [12,13,14]. Most of these studies focused on low-elevation hosts. However, few studies have explored how human skin microbiota adapts to extreme high-elevation environments. 

Qinghai–Tibet Plateau (QTP) is considered the highest plateau in the world with an average elevation of 3000–5000 m above sea level (mASL), and is also called “the roof of the world”. Human population on the QTP has exceeded 12 million since 2006 [15], and their physiology and immunity may be influenced by the extreme high-altitude environments. More narrowly, at the harsh high-elevation environment, human skin derived from the QTP suffers diverse extreme environmental conditions, including low pressure, low temperature, high ultraviolet radiation (UVR) intensity, and hypoxia [16,17]. Thus, these harsh environmental factors may exert tremendous selection forces on skin microbiotas, and thus influence the diversity of skin microbial communities. A decrease in skin microbial diversity is associated with several skin diseases (i.e., atopic dermatitis) in some clinical cases [18]. It is thus important to explore the skin microbial diversity patterns along environmental gradients. Several studies have reported the elevational diversity patterns of skin microbial communities in different host species [9,19,20]. However, skin microbial diversity patterns of different hosts follow distinct, changing patterns with elevation. For example, alpha diversity of salamander increased with elevation [9], while the skin microbiotas of Coqui frogs (*Eleutherodactylus coqui*) showed no manifest diversity patterns with altitude [19]. One recent study found that high-elevation humans and pigs had less skin microbial diversity than those at low-elevation, based on two limited elevation ranges (3750–3861 mASL and 319–1421mASL) [20], and the authors obtained skin samples for only one body site from two very close villages on the QTP. As a result, it is indispensable to explore human skin microbiota diversity patterns across multiple body sites or more extensive elevation gradients. 

In recent years, ecological networks have been popular in microbial ecology research. Network organization is important to understand community stability and ecosystem services. For example, the collapse of a microbial network structure is linked with seborrheic dermatitis [21]. Microbial networks consist of nodes and edges. Nodes are generally the species or other taxonomic units, and edges are the links (or correlations) between different species. Ecological networks can describe the co-occurrence patterns among microbes in microbial communities. Positive links between nodes indicates the cooperation or mutual benefit between microbial species, while negative links represent the competition or exclusion between species [22]. Network topological features may also reveal valuable biological information. For example, the network degree and density may reflect the complexity of interspecific interactions [23]. High modularity values represent a high degree of niche differentiation among species, and also weak microbial interactions [24,25]. Thus, using the network analysis can be used to uncover system-level ecological features in microbial communities. To date, few studies have compared the network topological characteristics of human skin microbiotas between elevations. 

Previous studies mainly investigated the composition and diversity of skin microbiota across different host species or environments. Few studies have explored the community assembly processes of skin microbial communities. Microbial community assembly processes consist of stochastic and deterministic processes [26,27]. Understanding community assembly processes may help to understand whether the microbial community can be regulated or predicted by external environmental factors. If one ecological community is primarily impacted through stochastic processes, then the variation trend of the community composition is unpredictable, uncertain, or non-directional [28]. In contrast, if one community is mainly controlled by deterministic processes, then the composition and diversity of the microbiota is predicted and directional, and can also be mediated by environmental factors [29]. In particular, the ecology theory from Vellend (2010) supposes that one community diversity is shaped by four main processes, including dispersal, drift, speciation, and selection [30]. Generally, the speciation is not considered at a short time or evolutionary scale. Consequently, ecologists have only focused on the processes selection, drift, and dispersal [31]. Stegen et al. (2013) further divided these three processes into variable selection, homogeneous selection, dispersal limitation, homogenizing dispersal, and ecological drift. The process homogeneous selection denotes that a coincident selective force, among local scales (i.e., the same pH among different soil samples), leads to similar community composition. Variable selection represents differences of the selective environment among local scales (i.e., different salinity along environmental gradients), which causes differences in community composition. The ecological significance of homogenizing dispersal is that the broad dispersal rate leads to similar community composition among local scales. The dispersal limitation signifies that a limited dispersal rate causes divergence in community composition. Ecological drift is derived from stochastic changes (i.e., birth or death) in population sizes. Selection (namely, variable selection and homogeneous selection) belongs to deterministic processes, but dispersal (including dispersal limitation and homogenizing dispersal) and drift belong to stochastic processes [32]. Understanding the community assembly processes of human skin microbiotas along elevation gradients will help us regulate and predict changes in the microbial community composition and diversity in order to improve or maintain host health under extreme environments. 

In this study, we explored the community diversity, predicted functions, network interactions, and assembly processes of human skin (including forehead, opisthenar, and palm) microbiotas along seven elevation gradients from 501 to 3431 mASL. We hypothesized that alpha and beta diversity patterns at high elevations were different from those at low elevations due to the filtering and selection of high-altitude environments. First, we tested which skin microbes could adapt well to high-elevation environments. Second, we evaluated whether the alpha diversity decreased while beta diversity of skin microbiotas increased with elevation. Third, we assessed whether there existed differences in network topological features between high- and low-elevation human skin microbial communities. Last, we want to know whether high-elevation human skin microbiotas had more deterministic processes compared with low-elevation microbiotas. 

## 2. Materials and Methods

### 2.1. Volunteer Recruitment and Sample Collection

Sample collection date was between October 22 and November 2 in the autumn of 2016. We recruited experimental volunteers for skin microbiota research. The criteria was that volunteers must be native adult individuals who have not left their homes for at least five years, and also have not had any skin diseases, antibiotics, or related drug use. No bathing or washing in each participant was done for at least 12 h before sample collection began. All participating subjects were of Han nationality. Finally, five adult subjects participated in our study in each sampling site. A total of 105 skin samples from 35 human participants were obtained from seven different elevation sites, including Chengdu (501 m), Xining (2298 m), Xingquan (2690 m), Guinan (3110 m), Hacheng (3150 m), Riyuexiang (3271 m), and Zeku (3431 m). Skin samples were collected using a wet sterile cotton swab from three different body sites, including the forehead, palm, and opisthenar. More concretely, the sampling area was 5 × 5 cm, and the time that the cotton wiped the skin was approximately 60 s. The cotton swaps collected were put into 2 mL sterile micro-centrifugal tubes, and stored in a –20 °C portable refrigerator immediately. To estimate the UVR during the sampling sites, we measured the UVR intensity at approximately 11:00–13:00 using an ultraviolet meter (UV-340A, spectrum range 290–390 nm, LUTRON, Taiwan). The average UVR values of all elevation sites ranged from 53 to 3785 uW/cm^2^ in the current study. All samples were finally transferred to our lab within 24 h. The information from each sample was recorded in Table S1. 

All experimental procedures were performed in compliance with the Ethics Committee of the Chinese Academy of Sciences (CAS-NWIPB-2016-137, approved on August 25, 2016). In addition, written informed consent was obtained from all volunteers in this study and was submitted to the related ethics committee. Notably, sample collection and experimental procedures sternly followed the related guidelines.

### 2.2. DNA Extraction and High-Throughput Sequencing

We extracted skin microbiota DNA through Soil Ezup genomic DNA extraction kits (Shanghai Sangon Biotech, China). Briefly, the detailed information of DNA extraction, polymerase chain reaction (PCR) amplifications, and gel extraction was described in our previous study [33,34,35]. Finally, an Illumina Miseq platform (Reagent Kit V2, Novogene, Beijing, China) with 2 × 300 cycles was used for sequencing the pooled amplicons. 

### 2.3. Bioinformatics Analysis

QIIME 1.9.0 was used to process the raw 16S rRNA gene sequencing data based on online commands (http://qiime.org/tutorials/tutorial.html), based on the Environmental Microbiome and Bioinfomatic Analysis Platform of the School of Public Health in Lanzhou University. Raw sequences were demultiplexed to each sample according to unique barcodes. Two original fastq sequences for each sample were joined through the FLASH-1.2.8 assembled software [36]. The reads that had a length of less than 300 bp, or included ambiguous bases, or possessed an average base quality score <Q30 were removed from the subsequent analysis. The remaining sequences were then subjected to a chimera test according to the Uchime algorithm [37]. Thereafter, we clustered OTUs (operational taxonomic units) at a 3% sequence dissimilarity based on Uclust [38]. Those OTUs that had the highest sequence number were picked as representative sequences, and were classified using the RDP (Ribosomal Database Project) classifier [39]. Those OTUs that were not identified as bacteria were removed. In addition, those OTUs that contained only one sequence were also rejected. To analyze different samples using the same sequencing depth, all samples were re-sampled into the same sequence number (15,596 sequences). Taxonomic compositions of skin microbiota samples were evaluated at phylum or genus level. The alpha diversity values (i.e., observed OTUs and Shannon diversity) were produced in QIIME, and the rarefaction curves of observed OTUs were produced. To evaluate beta diversity values, the two distance matrices, Jaccard and Bray–Curtis were calculated through QIIME. Notably, Jaccard distance considered the presence/absence of each OTU [40], but the Bray–Curtis distance matrix computed was dependent on each OTU abundance or percentage composition [41]. To understand the difference of skin microbiome across elevations and body sites, the non-metric multidimensional scaling (NMDS) plots of the two above matrices were visualized using Originlab 2018 (Originlab, Northampton, USA). 

### 2.4. Statistical Analysis

To understand the relative contributions of different factors on human skin microbiota, we evaluated whether the skin microbiota structures were significantly distinct across elevations based on PERMANOVA (permutational multivariate analysis of variance) [42] using the procedure ‘adonis’ in the R ‘vegan’ package. The model variables included elevation, individual, gender, age, height, weight, and body site (forehead, palm, and opisthenar). In each body site, one-way analysis of variance (one-way ANOVA) with a post hoc test was applied to uncover the differences of dominant phyla or genera between elevations. Core phyla or genera were defined as those genera that were present in all samples. Linear regression analysis was also carried out between elevation and core phyla (mean relative abundance >1%), alpha diversity (Shannon diversity and observed OTUs), or beta diversity (Jaccard and Bray–Curtis distance matrices). Spearman rank correlation analysis was used to detect the relationship between core genera (mean relative abundance >0.09%) and elevation. We defined those microbes that had a negative correlation with elevation as “elevation-sensitive microbes”, while those microorganisms that had a positive correlation with elevation that could be defined as “elevation-tolerant microbes”. *p* values in the related analysis were corrected using the FDR (false discovery rate) control. A partial mantel test was used to detect the effects of elevation on the skin microbiota after controlling host-related factors (including the individual, gender, age, height, weight, and body site) using the “mantel” procedure in the R “vegan” package. 

### 2.5. Co-Occurrence Patterns Analysis

The skin microbiotas of low-elevation areas from 501 to 2690 m showed clear structure separation from those from 3110 to 3431 m. Thus, we divided 105 samples into six groups, namely the group high-elevation forehead, low-elevation forehead, high-elevation opisthenar, low-elevation opisthenar, high-elevation palm, and low-elevation palm. Ecological network analysis was able to reveal the co-occurrence patterns between different microorganisms. To get rid of rare OTUs, those OTUs with a mean relative abundance less than 0.01% across all samples were removed. The Spearman rank correlation coefficients were calculated between two OTUs. *p*-values of correlation analysis were adjusted based on the Benjamini and Hochberg FDR controlling methods [43]. Based on the correlation coefficients and FDR-adjusted *p*-values, the meta-community network was constructed using the weighted correlation network analysis (WGCNA) package. The selected cutoff of *p*-values (FDR-adjusted) was 0.001, and the threshold of correlation coefficients was 0.77, using the methods that were dependent on the random matrix theory [44]. Each node in the network represents one OTU, and each edge that connects two nodes represents the correlation between OTUs. Network topological features were obtained with the “igraph” package. All the samples were then divided into six groups. Sub-network images of each group were visualized using the Gephi 0.9.2 (https://gephi.org/). 

To characterise the network topology, we calcualed four node-level topological features (i.e., closness centrality, node degree, betweeness centrality, and eigencentrality) and six network-level topological features (i.e., nodes, links, cluster number, average degree, graph density, and modularity) for each sub-network. A Wilcoxon rank-sum test was applied to determine the differences of node- and network-level topological features between groups. Those microbes with a higher node degree (more than 100) and lower betweenness centrality (less than 5000) values in networks were regarded as “keystone species” [45]. 

### 2.6. Community Assembly Processes Analysis 

To test which ecological processes may govern bacterial community assembly across groups, we used the methods of Stegen et al. (2013) to calculate the potential ecological processes, including the variable or homogeneous selection, dispersal limitation, homogenizing dispersal, and undominated processes (or called as “ecological drift” in [46]. To quantify community ecological processes, phylogenetic ecological diversity of bacterial communities was computed between any two samples in each group. The package “picante” in R was used to calculate the weighted beta nearest taxon index (β-NTI) parameters. The integration of Bray–Curtis-based Raup–Crick (RC_bray_) and β-NTI was applied to infer the relative contributions of the above processes dominating the skin microbiota. If the values of β-NTI were >2 or <−2, then this signified that the community turnover was modulated by the variable or homogeneous selection, respectively. In addition, if −2 < β-NTI < 2 as well as RC_bray_ > 0.95 or < −0.95, this indicated that the community composition was regulated by dispersal limitation or homogenizing dispersal, respectively. Lastly, if 2 < β-NTI < 2 and −0.95 < RC_bray_ < 0.95, this indicated that the community diversity was influenced by undominated processes or ecological drift [46]. Variable selection and homogeneous selection belonged to deterministic processes, yet dispersal (namely, dispersal limitation and homogenizing dispersal) processes and drift belong to stochastic processes [32]. Thus, the stochastic and deterministic processes of each group were also calculated. 

### 2.7. Function Prediction of Skin Microbiota

We used PICRUSTv1.0.0 [47] to predict the abundances of gene functions according to the OTU abundances. Then, we calculated the mean relative abundance of gene functions at level 3 within each elevation. The Bray–Curtis distance matrix was produced based on predicted gene functions using QIIME. For each skin site, analysis of similarity (ANOSIM) was also applied to uncover the difference between high- and low-elevation skin microbial functional profiles. The difference of gene functions at level 3 between high- and low-elevation sampling sites was calculated using the custom QIIME script “*otu_category_significance.py*”. *p* values were corrected using the Bonferroni methods. Only those gene functions between two groups with *p* < 0.01 were shown. 

## 3. Results

### 3.1. Overall Composition of Human Skin Microbiota 

**Figure 1:**
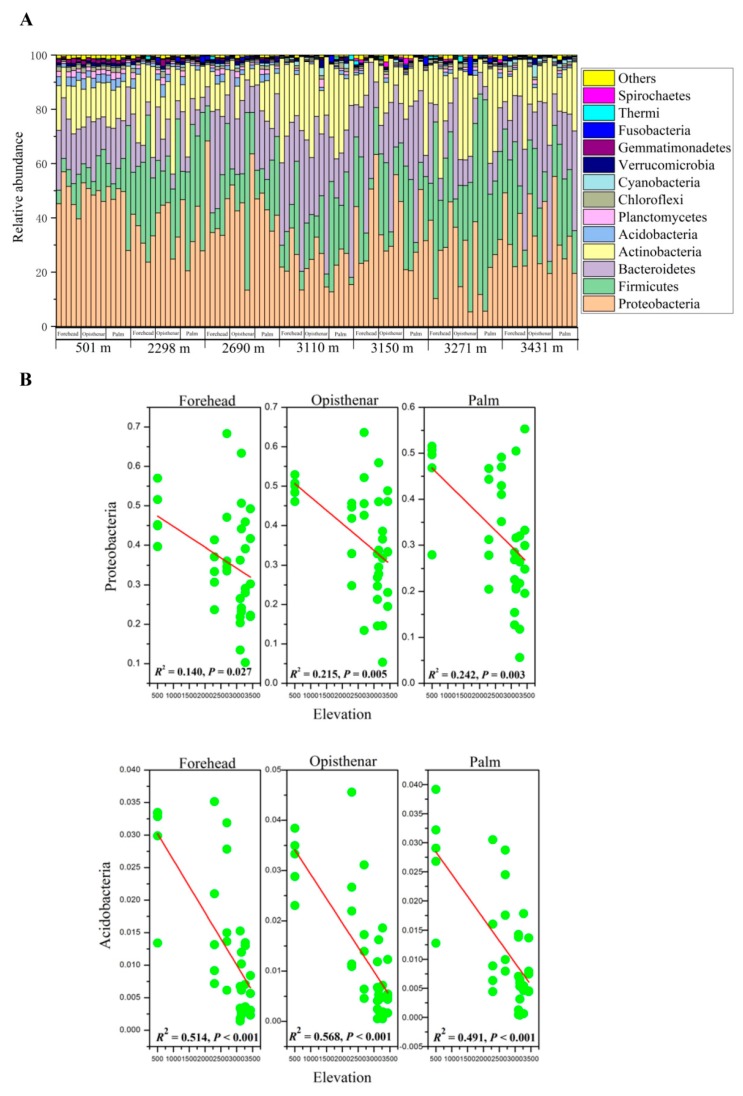
Skin community composition of each sample at the phylum level across elevations and body sites (**A**) Only those phyla with a mean relative abundance more than 0.1% were shown. Additionally, the relationship between dominant phyla and elevation (**B**) Only those phyla (mean relative abundance >1%) that were significantly correlated with elevation were shown.

After removing low-quality sequences, chloroplasts, chimeras, and singleton sequences, high-quality reads (1,945,102) were obtained from 105 human skin samples (mean 18,525 reads per sample, max = 19,605, min = 15,596, and *SD* = 765). In the current study, a total of 36,539 OTUs were identified and assigned to 58 phyla, 204 classes, 421 orders, 734 families, and 1593 genera. Human skin microbiota was dominated by the five most dominant bacterial phyla, including Proteobacteria (mean relative abundance = 34.87%), Firmicutes (21.99%), Bacteroidetes (20.31%), Actinobacteria (16.92%), and Acidobacteria (1.28%), and these phyla accounted for approximately 95% of the total sequences. Other rare phyla with a mean relative abundance <1% included Planctomycetes, Chloroflexi, Cyanobacteria, Verrucomicrobia, Gemmatimonadetes, Fusobacteria, Thermi, and Spirochaetes. The skin community composition of each sample is shown in Figure 1A. At the genus level, skin microbiota mainly consisted of *Chryseobacterium* (10.37%), *Acinetobacter* (6.12%), *Enhydrobacter* (4.58%), *Staphylococcus* (3.62%), and *Streptococcus* (3.35%). Other bacterial genera (mean relative abundance >1%) included *Corynebacterium*, *Lactobacillus*, *Ruminococcus*, *Planomicrobium*, *Lactococcus*, *Lysobacter*, *Cellvibrio*, *Prevotella*, *Micrococcus*, and *Luteimonas*. 

Core phyla included Proteobacteria, Firmicutes, Bacteroidetes, Actinobacteria, Acidobacteria, Planctomycetes, Chloroflexi, Verrucomicrobia, and Thermi, and these taxa were present in all samples. The total relative abundance of these phyla ranged from 92.8% to 99.9%. In addition, core genera consisted of 37 genera, such as *Acinetobacter*, *Chryseobacterium*, *Planomicrobium*, *Corynebacterium*, *Ruminococcus*, and *Prevotella*. The total abundance of these genera accounted for 13.8%–90.4% for each sample. 

### 3.2. Elevation-Sensitive and Elevation-Tolerant Microbes 

**Figure 2:**
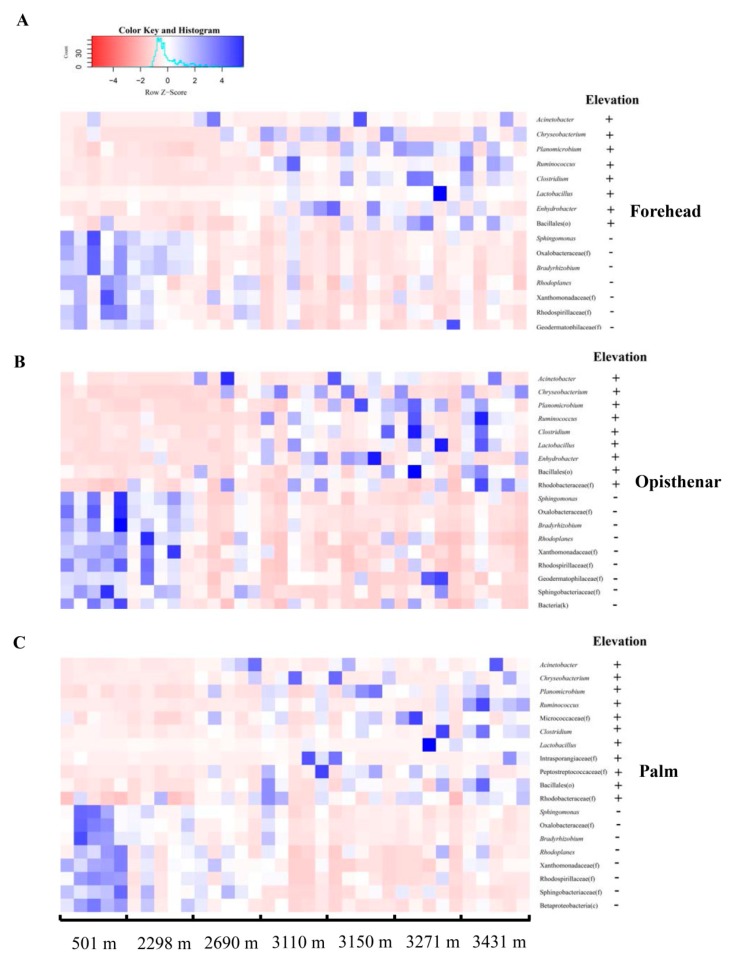
Spearman correlation between core genera (mean relative abundance >0.09%) of the forehead (**A**), opisthenar (**B**), and palm (**C**) and elevation. “+” means positive correlation, “-” means negative correlation.

We detected the correlation between major core taxa phyla (mean relative abundance >1%) or genera (mean relative abundance >0.09%) and elevation. Those microbes that had a positive correlation with elevation were defined as “elevation-tolerant” microbes, while those microorganisms that had a negative correlation with elevation were defined as “elevation-sensitive” microbes. Linear regression analysis showed that the total abundance of core phyla had no significant associations with elevation (all *p* > 0.05). Proteobacteria and Acidobacteria decreased with increasing elevation regardless of the body site (Figure 1B), indicating that members of these two phyla were elevation-sensitive microbes. However, Firmicutes, Bacteroidetes, and Actinobacteria of the forehead, opisthenar, or palm had no significant correlations with elevation (all *p* > 0.05). At the genus level, the total abundance of core genera was positively correlated with elevation (Figure S1). Elevation-tolerant microbes of forehead, opisthenar, and palm consisted of 8, 9, and 11 genera, respectively (Figure 2). Among these microbes, the shared elevation-tolerant genera in the three body sites included *Acinetobacter*, *Chryseobacterium*, *Planomicrobium*, *Ruminococcus*, *Clostridium*, *Lactobacillus,* and Bacillales (one unknown genus). In contrast, elevation-sensitive microbes of the forehead, opisthenar, and palm consisted of seven, nine, and eight genera, respectively (Figure 2). The shared elevation-sensitive microbes in the three body sites were affiliated with *Sphingomonas*, *Bradyrhizobium*, *Rhodoplanes*, Oxalobacteraceae, Xanthomonadaceae, Rhodospirillaceae, and Geodermatophilaceae. 

### 3.3. Alpha and Beta Diversity Patterns of Skin Microbiotas Along Elevational Gradients 

**Figure 3:**
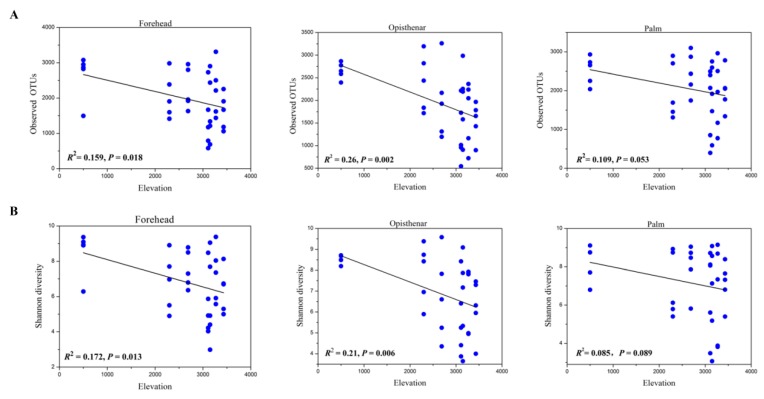
Linear regression relationship between alpha diversity values and elevation across body sites. (**A**) The relationship between observed OTUs and elevation on forehead, opisthenar and palm; (**B**) the relationship between Shannon diversity and elevation on forehead, opisthenar and palm.

Rarefaction curves of observed OTUs for each sample were produced at the OTU level (Figure S2). The curves of most of the samples reached close to the plateau, indicating that our sequencing depth was able to capture most bacterial species of human skin microbiota. While the Shannon diversity values showed a weak downward trend with elevation for the palm microbiota, most of the alpha diversity values (including observed OTUs and Shannon diversity) of human skin microbiota significantly decreased with increasing elevation (Figure 3). The fitting curve slope of opisthenar microbiota was more stepper, indicating that opisthenar microbial diversity was more influenced by elevation. Two-way ANOVA analysis showed that elevation influences the observed OTUs (*F* = 4.660, *p* < 0.001) or Shannon diversity (*F* = 3.476, *p* = 0.004) of human skin microbiota, while body sites or the interaction between elevation and body site had no significant effects shaping the alpha diversity values (all *p* > 0.05). In addition, UV was negatively correlated with Shannon diversity (Spearman *R* = −0.332, *p* < 0.001) or observed OTUs (*R* = −0.343, *p* < 0.001) of human skin microbiotas. 

**Figure 4:**
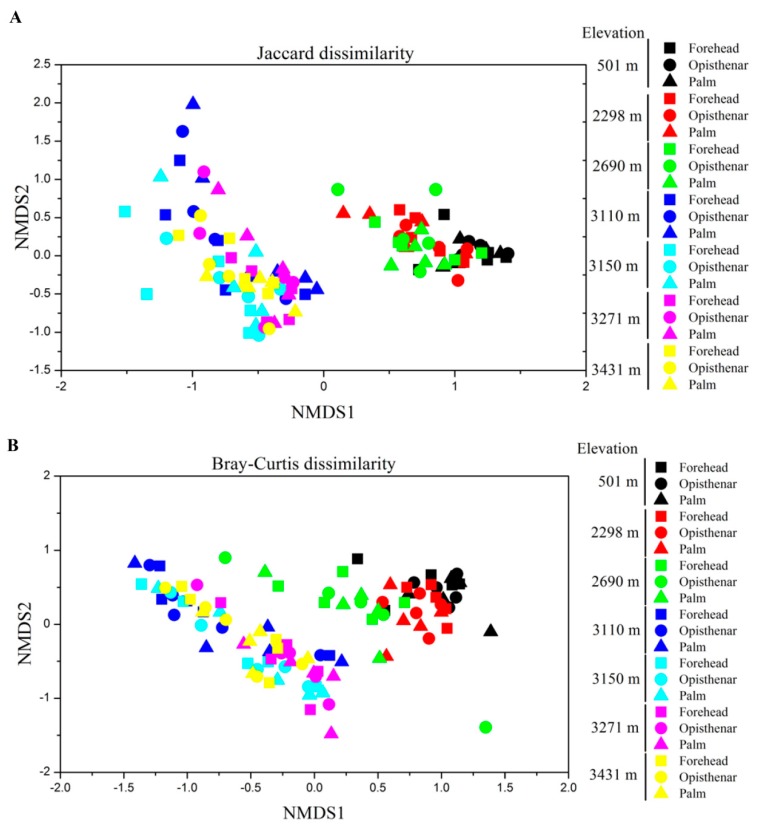
Non-metric multidimensional scaling (NMDS) plots showing the difference of human skin microbiota across elevations and body sites at operational taxonomic units (OTU) level based on (**A**) Jaccard and (**B**) Bray–Curtis distances.

The non-metric multidimensional scaling (NDMS) plots based on the Jaccard and Bray–Curtis matrices showed that the human skin microbiota structure had significant differences across elevations (Figure 4). The skin microbiotas of different body sites at the same elevation clustered together, indicating that the body site was a less important factor than elevation. PERMANOVA analysis found that skin microbiotas were mainly impacted by the individual (Jaccard *R*^2^ = 0.285, *p* < 0.001; Bray–Curtis *R*^2^ = 0.443, and *p* < 0.001) and elevation (Jaccard *R*^2^ = 0.059, *p* < 0.001; Bray–Curtis *R*^2^ = 0.149, and *p* < 0.001), followed by gender (Jaccard *R*^2^ = 0.056, *p* < 0.001; Bray–Curtis *R*^2^ = 0.101, and *p* < 0.001), age (Jaccard *R*^2^ = 0.016, *p* < 0.001; Bray–Curtis *R*^2^ = 0.082, and *p* < 0.001), height (Jaccard *R*^2^ = 0.014, *p* = 0.002; Bray–Curtis *R*^2^ = 0.023, and *p* < 0.001) and weight (Jaccard *R*^2^ = 0.011, *p* = 0.018; Bray–Curtis *R*^2^ = 0.021, and *p* < 0.001). The body site had a weak impact in shaping human skin microbiota based on the Bray–Curtis distance matrix (*R*^2^ = 0.015, *p* = 0.003), while there were no significant effects based on the Jaccard distance matrix (*R*^2^ = 0.018, *p* = 0.107). In addition, the skin microbiotas at low-elevation areas from 501 to 2690 m showed clear structure separation from those at high-elevation regions from 3110 to 3431 m. PERMANOVA confirmed that skin microbiotas between the high and low-elevation areas had significantly different community structures (Jaccard *R*^2^ = 0.079, *p* < 0.001; Bray–Curtis *R*^2^ = 0.179, *p* < 0.001). Notably, even after controlling host-related factors (including individual, gender, age, height, weight, and body site), elevation was still a significant factor that influenced human skin microbiota (partial mantel test r = 0.087, *p* < 0.001) based on the Bray–Curtis distance. 

**Figure 5:**
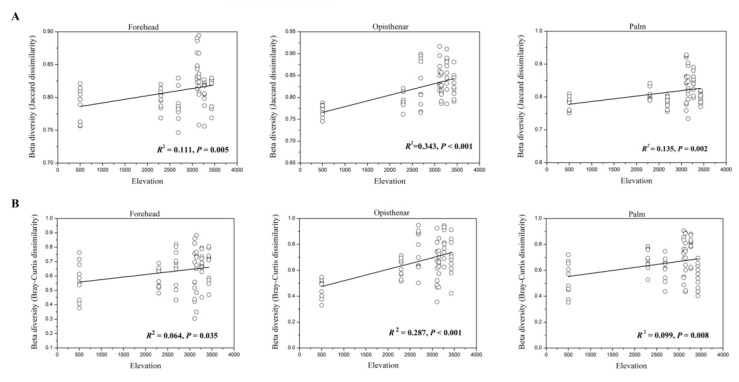
Beta diversity (Jaccard and Bray–Curtis dissimilarities within each elevation, **A**,**B**) values of human skin microbiota were significantly correlated with elevation (all *p* values < 0.05).

Interestingly, we found that beta diversity values increased with elevation in different body sites based on the Jaccard or Bray–Curtis dissimilarity matrix (all *p* < 0.05, Figure 5). In other words, the community dissimilarity between individuals was more different with increasing elevation. 

### 3.4. The Differences of Predicted Gene Functions between High and Low-Elevation Skin Microbiotas

A principal coordinate analysis (PCoA) plot based on the Bray–Curtis dissimilarity of predicted metagenomes at level 3 of KEEG (Kyoto Encyclopedia of Genes and Genomes) showed that forehead, opisthenar, and palm microbiota at high-elevation regions had different gene functional profiles than those at low-elevation regions (ANOSIM *r* =0.126, *p* < 0.01, Figure S3). ANOSIM analysis also showed that the individual, elevation, gender, host age, and weight significantly influenced the functions of skin microbiotas (all *p* < 0.05, Table S2). In particular, we compared the specific gene functions at level 3 for each skin site between high and low-elevation regions (Table S3). Those gene functional pathways involved in alanine, aspartate, and glutamate metabolism, and vitamin B6 metabolism were always enriched in the high-elevation sites, regardless of skin sites. In contrast, a total of eight shared gene functional pathways were more abundant in the low-elevation regions, these functional pathways included melanogenesis, glycan bindng proteins, neuroactive ligand-receptor interaction, pancreatic secretion, sesquiterpenoid biosynthesis, VEGF signaling pathway, CAM ligands, and ECM-receptor interaction (Table S3).

### 3.5. Co-Occurrence Patterns of Different Skin Subcommunities 

**Figure 6:**
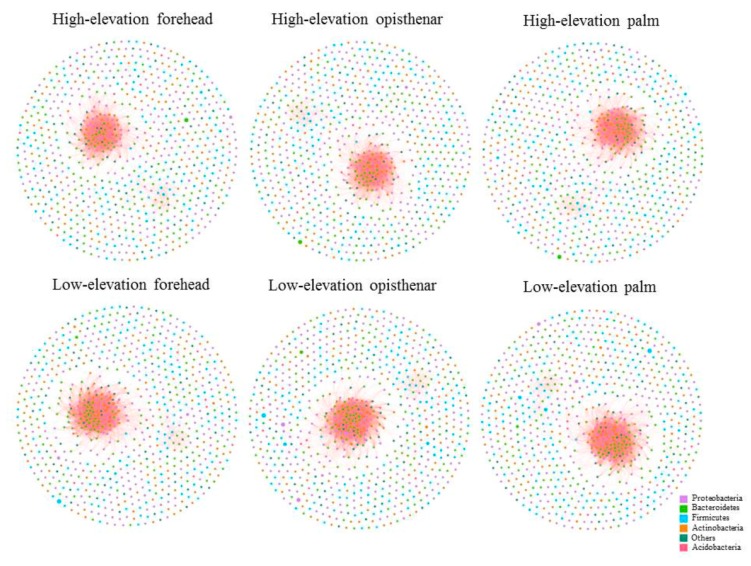
Different co-occurrence subnetworks of high-elevation and low-elevation human skin microbiotas based on a correlation analysis. A connection means a strong (Spearman’s *R* > 0.77) and significant (FDR-corrected *p* < 0.001) correlation. Abbreviation, FDR = False discovery rate.

**Figure 7:**
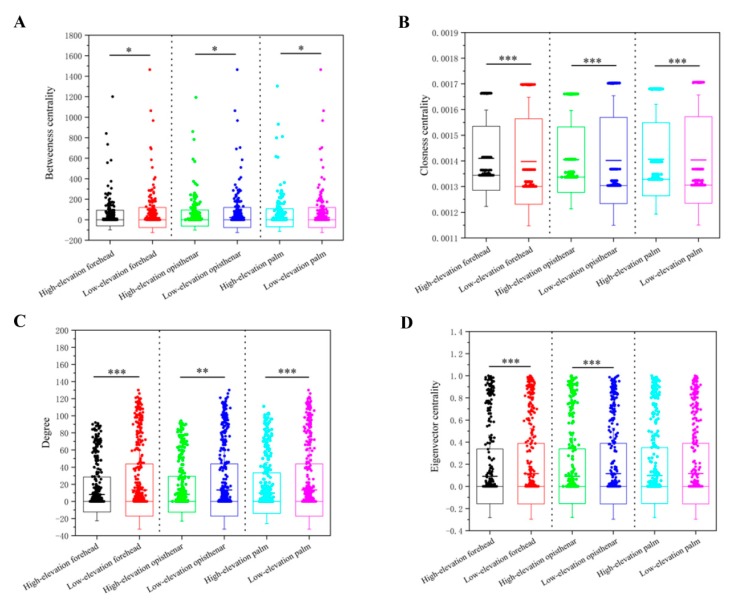
Four node-level topological features of high-elevation and low-elevation human skin microbiotas specifically the betweenness centrality (**A**), closeness centrality (**B**), degree (**C**), and eigenvector centrality (**D**). All values were significantly different between high-elevation and low-elevation based on the Wilcoxon rank sum tests (*p* < 0.05).

A meta-community ecological network was constructed based on the spearman correlations, and then six different sub-networks were split. These networks included the six groups, namely high-elevation forehead, low-elevation forehead, high-elevation opisthenar, low-elevation opisthenar, high-elevation palm, and low-elevation palm (Figure 6). The bacterial networks of high-elevation skin had less nodes and links (all links are positive) than those of low-elevation skin regardless of skin locations (Table S1). In addition, we also compared four node-level topological features of different subnetworks, including the betweenness centrality, closeness centrality, degree, and eigenvector centrality (Figure 7). The values of degree, betweenness centrality, and eigenbector centrality were significantly lower (*p* < 0.05) for high-elevation bacterial subnetworks than for low-elevation bacterial subnetworks with the exception of palm networks. This trend suggests that microbial taxa at the low-elevation were more located in the central positions within the corresponding network than those at the high-elevation. In other words, the bacterial taxa at the high-elevation were more located in the peripheral position in networks than those at the low-elevation. However, the values of closeness centrality at the high-elevation were significantly higher (*p* < 0.05) than those at the low-elevation, indicating that the nodes in the high-elevation networks were closer than those in the low-elevation networks. 

To confirm our above results, we also compared the overall network-level features of different skin microbiota subnetworks (Table 1). The clustering coefficient, average degree, and graph density were all lower at the high-elevation regions than at the low-elevation areas, suggesting that high-elevation skin bacterial networks were more fragile and less connected than low-elevation bacterial networks. Interestingly, we found that the modularity value was higher at high-elevation than at the low-elevation, indicating that a higher level of nich differentiation but weaker microbial interactions for high-elevation skin bacterial subnetworks. 

To identify the features of keystone species in networks, we picked out 31 keystone species in skin microbial networks (Table S4). These keystone species were mostly affiliated with *Flavisolibacter*, *Kaistobacter*, *Catellatospora*, *Steroidobacter*, *Glycomyces,* and *Chitinophaga*. Most of these keystone species (24 out of 31) had abundances of less than 0.1%, and were not the abundant taxa in human skin microbiota. For instance, the abundance ranking of OTU177470 (belonging to *Flavisolibacter*) with the highest abundance in the pool of keystone species were only 55th in skin microbiota. 

### 3.6. Ecological Processes Governing the Assembly of Human Skin Microbiotas

**Figure 8:**
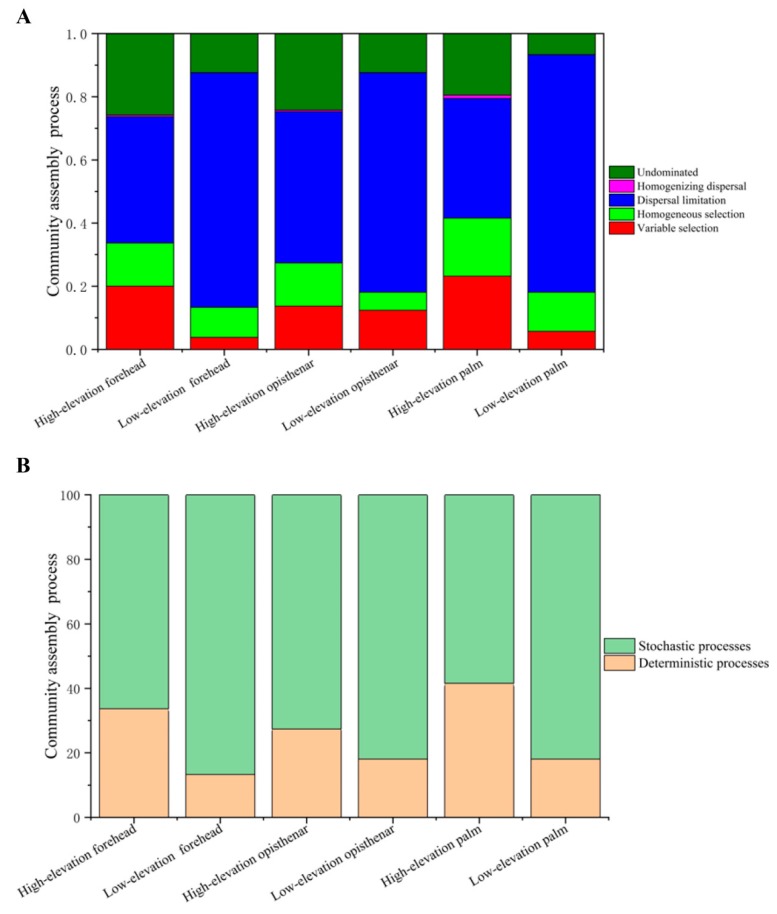
Summary of the contributions of the ecological processes that determine human skin microbiota assembly in different elevations. (**A**) The contributions of variable selection, homogeneous selection, dispersal limitation, homogenizing dispersal, and undominated in the assembly of human skin microbiota. (**B**) The relative contributions of deterministic processes and stochastic processes in human microbiota assembly.

To understand community assembly processes of human skin microbiota, we calculated which ecological processes governed the skin microbial communities between high and low-elevation regions (Figure 8). For forehead, opisthenar, or palm, the high-elevation skin microbiotas had more variable selection and homogeneous selection processes, but less dispersal limitation than the low-elevation skin microbiotas (Figure 8A). Variable selection and homogeneous selection belong to deterministic processes, while dispersal (dispersal limitation and homogenizing dispersal) and undominated processes belonged to stochastic processes. We found that human skin microbiotas were assembled by stochastic processes (58.4%–74.6%), but skin microbiota assembly at the high-elevation had more deterministic processes (or less stochastic processes) than that at the low-elevation (Figure 8B).

## 4. Discussion

Impacted by the immune system, host lifestyle, sanitary conditions, and environmental factors, human skin microbiome is associated with various diseases [48,49]. Thus, understanding skin microbiota is very important to explore the relationship between microbiotas and human health. Previous studies on skin microbiotas mainly focused on low-elevation humans and few reports have uncovered diversity patterns, network interactions, and assembly processes of human skin microbiotas on high-elevation humans. Using microbial ecology theory and network topological analysis, we found that elevation is a significant factor in shaping human skin microbiota diversity regardless of body sites. Alpha diversity values decrease with elevation, while beta diversity showed an opposite trend. Notably, high-elevation skin microbiota networks were more fragile than those at low-elevation areas, which possibly contribute to the higher incidence of microbiome-associated skin diseases in high-altitude regions. In addition, stochastic processes dominate the human skin microbiotas, but skin microbiotas at high elevation harbor more portion of deterministic processes than those at low-elevation, indicating that high-elevation environments may be conducive to the promotion of deterministic processes. These results greatly expand our understanding for human skin microbiota assembly under extreme environments. 

### 4.1. Elevation-Tolerant Microbes Indicate That the Adaptation of Skin Microbiota for Extreme High-Elevation Environment 

The relative abundance of elevation-tolerant microbes increased with increasing elevation. In this study, we found that *Acinetobacter*, *Chryseobacterium*, *Planomicrobium*, *Ruminococcus*, *Clostridium*, *Lactobacillus*, and Bacillales (one unknown genus) belonged to elevation-tolerant microbes regardless of human body sites, indicating that this genus in the human skin may possibly adapt well to harsh, cold, and high-UV environments at higher elevations. Interestingly, some elevation-tolerant microbes have been reported as extremophiles derived from high-elevation regions, indicating that the presence or increased abundance of these bacterial taxa is probably due to the exposure and selection of high-elevation environments. For example, several species of *Acinetobacter* have been isolated from extreme high-elevation lakes and wetlands [50,51]. These species can have a strong resistance to desiccation, starvation, cold, and UV. They also have efficient DNA damage repair ability when exposed to high UV [52]. While members of the genus Acinetobacter are strictly aerobic, they are able to form intracellular polymers (polyhydroxyalkanoates) under adverse environmental conditions (e.g., lack of oxygen) [53]. Thus, *Acinetobacter* should have broad adaptability capacity for extreme high-elevation environments. Notably, *Acinetobacter* is a common opportunistic pathogen in hospital, which may lead to various infections, such as pneumonia and bacteremia [54]. We speculate that a high abundance of this genus at high elevations may be associated with a higher risk of diseases. Future studies should uncover the functional role of *Acinetobacter* in human skin. 

In contrast, we found that shared elevation-sensitive microbes in different skin sites included *Sphingomonas*, *Bradyrhizobium*, *Rhodoplanes*, Oxalobacteraceae, Xanthomonadaceae, Rhodospirillaceae, and Geodermatophilaceae. These microbes may be very sensitive to extreme environments in high-elevation regions. For instance, some members of this genus will lose activation when exposed to a low UV dose of 40 mJ/cm^2^ [55]. Thus, the relative abundance of *Sphingomonas* decreased with increasing elevation, which is partly due to higher UV at high-elevation environments. *Bradyrhizobium* are genneral symbiotic bacteria associated with plants [56] and is rarely detected in human skin. Further studies should explore whether *Bradyrhizobium* is simply a native inhabitant or a transient microbe picked up from the environment. 

### 4.2. Alpha Diversity Decreases but Beta Diversity Increases for Skin Microbiota Along the Elevational Gradient

Generally, ecologists consider that one ecosystem that harbors more species diversity should be more stable, and is able to exhibit high levels of ecosystem functions and services [57,58]. While species can interact differently in different ecosystems, the evidence showed that high species diversity was able to provide more functional redundancy and buffer ecosystem functions against possible species loss or extinction [59,60] when facing environmental disturbance. In skin-microbe systems, a diverse skin microbiota may contribute a distinct set of enzymes for decomposing toxic substances, and also limit the overabundance of specific pathogenic bacteria associated with skin diseases by competing with them. On the contrary, a decrease in skin microbial diversity was associated with skin diseases [18]. Our results found that skin microbial diversity decreased with increasing elevation regardless of body sites, indicating that high-elevation humans are possibly linked to a higher prevalence of skin diseases. Our results are largely consistent with those of Zeng et al. (2017), who also found that alpha diversity values of high-altitude humans and pigs were lower compared to those at lower altitudes. High elevation is associated with extreme environmental conditions, such as high UV, which may kill microbes or restrain their growth, and lead to species loss. Our data also supported this viewpoint, because UV was negatively correlated with alpha diversity of human skin microbiotas. In addition to UV, those unmeasured factors, such as temperature and oxygen concentration may also influence skin bacterial diversity [61,62]. Interestingly, elevation had the greatest impact on opisthenar microbial diversity, followed by the forehead and palm. The possible reason is that opisthenar suffers from exposure to more light (or UV), while the Han Chinese often wear hats, and their forehead and palm receive less exposure to UV. Thus, the loss rate of bacterial species on opisthenar is higher. Nevertheless, body sites had no significant impacts in microbial alpha diversity, suggesting that human microbial diversity is relatively stable in different skin sites. 

Our data showed that the individual was the most important factor in determining human skin microbiota. The results were largely the same as previous reports [3,63], where it was also found that the composition of human individual skin microbiome was highly personalized. This microbiota variability is probably dependent on an individual trait, because different human individuals have discrepant host physiological characteristics. These findings suggest that individualized treatment is needed to regulate skin microbiomes in order to treat diseases. In addition to the individual, elevation is the second most important factor impacting community structure. We found that human skin community structures were significantly different across elevations, which is similar to other studies regarding skin microbial communities in humans, pigs, and amphibian [20,64]. In addition to UV, as mentioned above, other unmeasured environmental factors (e.g., air temperature) associated with elevation may influence the skin microbiota structure [65]. As UV and elevation was highly auto-correlative, we could not disentangle the relative contributions of each of these two factors on the skin microbiota structure. However, we found that elevation was still a significant factor that influenced human skin microbiota even after controlling host-associated factors, indicating that elevation was able to impact skin microbiomes.

Beta diversity values (including Jaccard and Bray-Curtis dissimlarities) of human skin microbiotas within each elevation also increased with increasing elevation, indicating that inter-individual skin microbial communities were more different at high-elevation regions. We listed two hypotheses for future verification. First, high-altitude environmental pressure may cause different physiological and immune responses for each human individual [66], thus probably indirectly increasing the difference of inter-individual skin microbiota. Second, environmental (i.e., building environments) microbes at different altitudes may also influence host skin microbiota, as it has been demonstrated that human homes may shape skin bacterial classes [67]. In addition to the difference of microbial community diversity, functional differences between high-elevation and low-elevation skin microbiotas also existed. These results indicated that elevation also significantly impacts the skin community functions. Notably, we found that vitamin B6 metabolism was always enriched in the high-elevation sites regardless of skin sites, suggesting that vitamin B6 metabolism is very important in high-elevation environments. Indeed, vitamin B6 was specifically efficient in quenching reactive oxygen species. Consequently, related results from different organisms found that reduced levels of vitamin B6 were linked with severe susceptibility to extreme environmental stress (oxidative, drought, and UV-B) [68]. Thus, the enriched vitamin B6 metabolism at high elevation may probably be one kind of functional adaptability for human skin microbiota. Interestingly, our results also showed that human skin microbiotas with more than 3000 m altitude had a significant structural or functional separation with less than 3000 m. The possible reason is that approximately 3000 m elevation is a demarcation line for obvious changes in human physiology [15], which may enormously influence the composition and function of skin microbiota.

### 4.3. High-Elevation Skin Microbiota Networks are More Fragile Than Those at Low-Elevation Areas 

Network analysis is able to reveal the complex inter-interactions of different bacterial species in microbial ecology study [25]. Here, the bacterial networks of high-elevation skin had less nodes and links than those of low-elevation skin regardless of skin locations, indicating that a high-elevation environment led to simpler skin bacterial networks. All links were positive under the network construction condition and the cutoff of correlation coefficients for nodes was 0.77, implying that the cooperation or mutual benefit probably plays a dominant role among human skin microbiota. Node-level topological features revealed significantly lower values of degree, betweenness centrality, and eigenbector centrality for high-elevation subnetworks than for low-elevation subnetworks, suggesting that bacterial taxa at the high-elevation were more located in the peripheral position in networks. For network-level topological features, different subcommunities uncovered less connection for bacterial taxa at high-elevation than those at low-elevation regardless of body sites. Thus, microbes had a more distant relationship and less influence on other co-occurrences at high-elevation bacterial networks. In other words, the microorganisms at the high-elevation networks had a more independent status. It has been found that the bacterial networks with less graph density and clustering coefficients in megacities were more fragile than non-megacities [48], and human population in megacities was associated with more skin related diseases. Similarly, our results showed the bacterial networks at high-elevation had less graph density and clustering coefficients than those at the low-elevation. Thus, high-elevation humans may likely be more susceptible to related disease risk. 

Our data showed that most of keystone species in networks had a low abundance and were relatively rare in skin microbiota. The most abundant keystone species OTU177470 was at the 55th position, suggesting that the keystone species in microbial networks were not necessarily the dominant microbes in human skin. These results were congruent with those in other ecosystems [22,69], which also found that rare species in microbial communities were keystone species. This ecological result was advantageous to maintain stable network structures, because networks may recruit keystone species from those rare taxa [70], which has a higher diversity compared with abundant microbes, rather than depending on the input of new bacteria or choosing limited abundant species. Nevertheless, the topological characteristics of our established networks were dependent on sequencing depth and correlation threshold, and a network construction method by retaining OTUs with >0.01% average relative abundance across all samples. This data processing method may probably impact the topological features of microbial networks. 

### 4.4. Stochastic Processes Dominate the Human Skin Microbiota, but High-Altitude Skin Microbiota Harbors More Deterministic Processes

While the estimation of ecological processes may be influenced by technical deficiencies, such as the difference of DNA extraction methods, PCR-bias, and sequencing errors, this method has been found to be effective and widely applied in analyzing the microbiota assembly processes of humans, pikas, fruit fly, and other environments [71,72,73,74]. Based on the phylogenetic analysis, we found that human skin microbiota was mainly assembled by stochastic processes, implying that individual skin microbial composition can be unpredictable, and stochastic factors (i.e., stochastic dispersal, birth, or death) may be responsible for the community assembly. Our results were not inconsistent with the previous study by Kim et al. (2018), who found human skin microbiotas are assembled primarily by niche-based processes (similar to deterministic processes) based on null model testing. The possible reason is that Kim et al. (2018) did not consider the phylogenetic relationship of bacterial species. Our results indicate that it is difficult to regulate skin microbial ecology to improve human health due to the stochastic assembly of human skin microbiota. 

We found that high-elevation human skin microbiotas had more fractions of deterministic processes regardless of skin locations, suggesting that elevation acts as strict environmental filters and cause the increase of ‘selection’ processes. Our data supported this inference, as demonstrated by the finding that more variable selection and homogeneous selection processes were detected in high-elevation humans. Environmental filtering is a pivotal determinant of ecological community assembly [75] and leads to phylogenetic clustering of closely related microorganisms and increase the deterministic processes in human skin. In addition, high-elevation environments may improve human immune response [66] and the related immunity may select or enrich specific bacteria that adapt to the extreme environment. Thus, deterministic processes increased in high-altitude humans. In addition, we found that high-altitude humans had less dispersal limitations than those at the low-elevation, implying that the former had less frequency of microbial transmission, probably due to less population density or physical contact on the QTP. Further studies should investigate which factors determine microbial community processes. 

## 5. Conclusions 

We are the first to study the diversity, function, interaction, and assembly of human skin microbiotas along a wide elevational gradient. Our results showed that specific human skin bacteria, such as *Acinetobacter,* might adapt to the cold, dry, and high-UV plateau environments. The individual is the most important factor in determining skin microbiota. Elevation is a significant factor that influences human skin microbiota, even after controlling host-related factors. Due to the filtering of high-altitude environments, the alpha diversity of human skin microbiotas decreased with elevation, but the beta diversity showed an opposite pattern. While stochastic processes dominate the human skin microbiota, high-altitude skin microbiota harbors more deterministic processes. Thus, it is quite difficult to regulate skin microbial ecology to improve human health due to the stochastic assembly of skin microbiota. The bacterial networks at high-elevation were more fragile than those at low-elevation. Consequently, high-elevation humans may likely be more susceptible to related disease risk. However, this study has a limited sample size, a larger number of samples may make our results more accurate. In addition, further studies should focus on the relationship between skin microbiota and human health. 

Nucleotide Sequence Accession Numbers: The original 16S rRNA gene sequencing data in this study were deposited at the European Nucleotide Archive by accession number PRJEB31799 (http://www.ebi.ac.uk/ena/data/view/ PRJEB31799). 

## Figures and Tables

**Table 1:** Network-level topological features of the bacterial subnetworks in human skin.

Group	Nodes	Edges	Clustering Coefficient	Average Degree	Graph Density	Modularity
High-elevation forehead	744	3018	0.7337	8.1129	0.0109	0.1697
Low-elevation forehead	769	5136	0.7443	13.3576	0.0174	0.1166
High-elevation opisthenar	748	3123	0.7269	8.3503	0.0112	0.1591
Low-elevation opisthenar	767	5130	0.7444	13.3768	0.0175	0.1130
High-elevation palm	753	3661	0.7276	9.7238	0.0129	0.1461
Low-elevation palm	766	5116	0.7444	13.3577	0.0175	0.1172

## Supplementary Materials

Supplementary materials can be found at https://www.mdpi.com/2076-2607/7/12/611/s1.

## References

[1] Chen Y.E., Fischbach M.A., Belkaid Y. (2018). Skin microbiota-host interactions. Nature.

[2] Moissl-Eichinger C., Probst A.J., Birarda G., Auerbach A., Koskinen K., Wolf P., Holman H.N. (2017). Human age and skin physiology shape diversity and abundance of Archaea on skin. Sci. Rep..

[3] Oh J., Byrd A.L., Park M., Program N.C.S., Kong H.H., Segre J.A. (2016). Temporal Stability of the Human Skin Microbiome. Cell.

[4] Belkaid Y., Tamoutounour S. (2016). The influence of skin microorganisms on cutaneous immunity. Nat. Rev. Immunol..

[5] Grice E.A. (2015). The intersection of microbiome and host at the skin interface: Genomic- and metagenomic-based insights. Genome Res..

[6] Findley K., Oh J., Yang J., Conlan S., Deming C., Meyer J.A., Schoenfeld D., Nomicos E., Park M., Sequencing N.I. (2013). Topographic diversity of fungal and bacterial communities in human skin. Nature.

[7] Grice E.A., Kong H.H., Conlan S., Deming C.B., Davis J., Young A.C., Program N.C.S., Bouffard G.G., Blakesley R.W., Murray P.R. (2009). Topographical and temporal diversity of the human skin microbiome. Science.

[8] Grice E.A., Segre J.A. (2011). The skin microbiome. Nat. Rev. Genet..

[9] Muletz Wolz C.R., Yarwood S.A., Campbell Grant E.H., Fleischer R.C., Lips K.R. (2018). Effects of host species and environment on the skin microbiome of Plethodontid salamanders. J. Anim. Ecol..

[10] Perez Perez G.I., Gao Z., Jourdain R., Ramirez J., Gany F., Clavaud C., Demaude J., Breton L., Blaser M.J. (2016). Body Site Is a More Determinant Factor than Human Population Diversity in the Healthy Skin Microbiome. PLoS ONE.

[11] SanMiguel A., Grice E.A. (2015). Interactions between host factors and the skin microbiome. Cell Mol. Life Sci..

[12] Grice E.A., Snitkin E.S., Yockey L.J., Bermudez D.M., Program N.C.S., Liechty K.W., Segre J.A., Mullikin J., Blakesley R., Young A. (2010). Longitudinal shift in diabetic wound microbiota correlates with prolonged skin defense response. Proc. Natl. Acad. Sci. USA.

[13] Nakatsuji T., Chiang H.I., Jiang S.B., Nagarajan H., Zengler K., Gallo R.L. (2013). The microbiome extends to subepidermal compartments of normal skin. Nat. Commun..

[14] Smeekens S.P., Huttenhower C., Riza A., van de Veerdonk F.L., Zeeuwen P.L., Schalkwijk J., van der Meer J.W., Xavier R.J., Netea M.G., Gevers D. (2014). Skin microbiome imbalance in patients with STAT1/STAT3 defects impairs innate host defense responses. J. Innate Immun..

[15] Wu T.Y. (2006). Challenges of plateau hypoxic environment to humans. J. Med. Res..

[16] Beall C.M. (2006). Andean, Tibetan, and Ethiopian patterns of adaptation to high-altitude hypoxia. Integr. Compar. Biol..

[17] Jablonski N.G., Chaplin G. (2010). Human skin pigmentation as an adaptation to UV radiation. Proc. Natl. Acad. Sci. USA.

[18] Kong H.H., Oh J., Deming C., Conlan S., Grice E.A., Beatson M.A., Nomicos E., Polley E.C., Komarow H.D., Program N.C.S. (2012). Temporal shifts in the skin microbiome associated with disease flares and treatment in children with atopic dermatitis. Genome Res..

[19] Hughey M.C., Pena J.A., Reyes R., Medina D., Belden L.K., Burrowes P.A. (2017). Skin bacterial microbiome of a generalist Puerto Rican frog varies along elevation and land use gradients. PeerJ.

[20] Zeng B., Zhao J., Guo W., Zhang S., Hua Y., Tang J., Kong F., Yang X., Fu L., Liao K. (2017). High-Altitude Living Shapes the Skin Microbiome in Humans and Pigs. Front. Microbiol..

[21] Park T., Kim H., Myeong N., Lee H., Kwack I., Lee J., Kim B., Sul W., An S. (2017). Collapse of human scalp microbiome network in dandruff and seborrheic dermatitis. Exp. Dermatol..

[22] Li H., Li T., Tu B., Kou Y., Li X. (2017). Host species shapes the co-occurrence patterns rather than diversity of stomach bacterial communities in pikas. Appl. Microbiol. Biotechnol..

[23] Li H., Li T., Berasategui A., Rui J., Zhang X., Li C., Xiao Z., Li X. (2017). Gut region influences the diversity and interactions of bacterial communities in pikas (*Ochotona curzoniae* and *Ochotona daurica*). FEMS Microbiol. Ecol..

[24] Li H., Li T., Li X., Wang G., Lin Q., Qu J. (2018). Gut Microbiota in Tibetan Herdsmen Reflects the Degree of Urbanization. Front. Microbiol..

[25] Faust K., Raes J. (2012). Microbial interactions: From networks to models. Nat. Rev. Genet..

[26] Stegen J.C., Lin X., Fredrickson J.K., Chen X., Kennedy D.W., Murray C.J., Rockhold M.L., Konopka A. (2013). Quantifying community assembly processes and identifying features that impose them. ISME J..

[27] Stegen J.C., Lin X., Konopka A.E., Fredrickson J.K. (2012). Stochastic and deterministic assembly processes in subsurface microbial communities. ISME J..

[28] Li S.P., Cadotte M.W., Meiners S.J., Pu Z., Fukami T., Jiang L. (2016). Convergence and divergence in a long-term old-field succession: The importance of spatial scale and species abundance. Ecol. Lett..

[29] Anderson K.J. (2007). Temporal patterns in rates of community change during succession. Am. Nat..

[30] Vellend M. (2010). Conceptual synthesis in community ecology. Q. Rev. Biol..

[31] Vellend M., Srivastava D.S., Anderson K.M., Brown C.D., Jankowski J.E., Kleynhans E.J., Kraft N.J.B., Letaw A.D., Macdonald A.A.M., Maclean J.E. (2014). Assessing the relative importance of neutral stochasticity in ecological communities. Oikos.

[32] Dini-Andreote F., Stegen J.C., van Elsas J.D., Salles J.F. (2015). Disentangling mechanisms that mediate the balance between stochastic and deterministic processes in microbial succession. Proc. Natl. Acad. Sci. USA.

[33] Li H., Li T., Yao M., Li J., Zhang S., Wirth S., Cao W., Lin Q., Li X. (2016). Pika gut may select for rare but diverse environmental bacteria. Front. Microbiol..

[34] Li H., Qu J., Li T., Li J., Lin Q., Li X. (2016). Pika population density is associated with composition and diversity of gut microbiota. Front. Microbiol..

[35] Li H., Li T., Beasley D.E., Hedenec P., Xiao Z., Zhang S., Li J., Lin Q., Li X. (2016). Diet Diversity Is Associated with Beta but not Alpha Diversity of Pika Gut Microbiota. Front. Microbiol..

[36] Magoc T., Salzberg S.L. (2011). FLASH: Fast length adjustment of short reads to improve genome assemblies. Bioinformatics.

[37] Edgar R., Haas B., Clemente J., Quince C., Knight R. (2011). UCHIME improves sensitivity and speed of chimera detection. Bioinformatics.

[38] Edgar R.C. (2010). Search and clustering orders of magnitude faster than BLAST. Bioinformatics.

[39] Wang Q., Garrity G.M., Tiedje J.M., Cole J.R. (2007). Naive Bayesian classifier for rapid assignment of rRNA sequences into the new bacterial taxonomy. Appl. Environ. Microbiol..

[40] Jaccard P. (1912). The distribution of the flora in the alpine zone. New Phytol..

[41] Bray J., Curtis J. (1957). An ordination of the upland forest communities of southern Wisconsin. Ecol. Monogr..

[42] McArdle B., Anderson M. (2001). Fitting multivariate models to community data: A comment on distance-based redundancy analysis. Ecology.

[43] Benjamini Y., Krieger A., Yekutieli D. (2006). Adaptive linear step-up procedures that control the false discovery rate. Biometrika.

[44] Luo F., Zhong J., Yang Y., Scheuermann R., Zhou J. (2006). Application of random matrix theory to biological networks. Phys. Lett..

[45] Xue Y., Chen H., Yang J.R., Liu M., Huang B., Yang J. (2018). Distinct patterns and processes of abundant and rare eukaryotic plankton communities following a reservoir cyanobacterial bloom. ISME J..

[46] Stegen J.C., Lin X., Fredrickson J.K., Konopka A.E. (2015). Estimating and mapping ecological processes influencing microbial community assembly. Front. Microbiol..

[47] Langille M.G., Zaneveld J., Caporaso J.G., McDonald D., Knights D., Reyes J.A., Clemente J.C., Burkepile D.E., Thurber R.L.V., Knight R. (2013). Predictive functional profiling of microbial communities using 16S rRNA marker gene sequences. Nat. Biotechnol..

[48] Kim H., Kim H., Kim J., Myeong N., Kim T., Park T., Kim E., Choi J., Lee J., An S. (2018). Fragile skin microbiomes in megacities are assembled by a predominantly niche-based process. Sci. Adv..

[49] Findley K., Grice E. (2014). The skin microbiome: A focus on pathogens and their association with skin disease. PLOS Pathog..

[50] Dib J., Motok J., Zenoff V.F., Ordonez O., Farias M.E. (2008). Occurrence of resistance to antibiotics, UV-B, and arsenic in bacteria isolated from extreme environments in high-altitude (above 4400 m) Andean wetlands. Curr. Microbiol..

[51] Ordonez O.F., Flores M.R., Dib J.R., Paz A., Farias M.E. (2009). Extremophile culture collection from Andean lakes: Extreme pristine environments that host a wide diversity of microorganisms with tolerance to UV radiation. Microb. Ecol..

[52] Albarracin V.H., Pathak G.P., Douki T., Cadet J., Borsarelli C.D., Gartner W., Farias M.E. (2012). Extremophilic Acinetobacter strains from high-altitude lakes in Argentinean Puna: Remarkable UV-B resistance and efficient DNA damage repair. Orig. Life Evol. Biosph..

[53] Kung S.S., Chuang Y.C., Chen C.H., Chien C.C. (2007). Isolation of polyhydroxyalkanoates-producing bacteria using a combination of phenotypic and genotypic approach. Lett. Appl. Microbiol..

[54] Bergogne-Berezin E., Towner K.J. (1996). Acinetobacter spp. as nosocomial pathogens: Microbiological, clinical, and epidemiological features. Clin. Microbiol. Rev..

[55] Sun W., Liu W., Cui L., Zhang M., Wang B. (2013). Characterization and identification of a chlorine-resistant bacterium, Sphingomonas TS001, from a model drinking water distribution system. Sci. Total. Environ..

[56] Antoun H., Beauchamp C.J., Goussard N., Chabot R., Lalande R. (1998). Potential of Rhizobium and Bradyrhizobium species as plant growth promoting rhizobacteria on non-legumes: Effect on radishes (*Raphanus sativus* L.). Molecular Microbial Ecology of the Soil.

[57] Margalef R. (1963). On certain unifying principles in ecology. Am. Nat..

[58] McNaughton S.J. (1977). Diversity and stability of ecological communities: A comment on the role of empiricism in ecology. Am. Nat..

[59] Bannar-Martin K.H., Kremer C.T., Ernest S.K.M., Leibold M.A., Auge H., Chase J., Declerck S.A.J., Eisenhauer N., Harpole S., Hillebrand H. (2018). Integrating community assembly and biodiversity to better understand ecosystem function: The Community Assembly and the Functioning of Ecosystems (CAFE) approach. Ecol. Lett..

[60] Louca S., Polz M.F., Mazel F., Albright M.B.N., Huber J.A., O’Connor M.I., Ackermann M., Hahn A.S., Srivastava D.S., Crowe S.A. (2018). Function and functional redundancy in microbial systems. Nat. Ecol. Evol..

[61] Murillo N., Raoult D. (2013). Skin microbiota: Overview and role in the skin diseases acne vulgaris and rosacea. Future Microbiol..

[62] Mathieu A., Vogel T.M., Simonet P. (2014). The future of skin metagenomics. Res. Microbiol..

[63] Flores G., Caporaso J., Henley J., Rideout J., Domogala D., Chase J., Leff J., Vázquez-Baeza Y., Gonzalez A., Knight R. (2014). Temporal variability is a personalized feature of the human microbiome. Genome Biol..

[64] Medina D., Hughey M.C., Becker M.H., Walke J.B., Umile T.P., Burzynski E.A., Iannetta A., Minbiole K.P.C., Belden L.K. (2017). Variation in Metabolite Profiles of Amphibian Skin Bacterial Communities Across Elevations in the Neotropics. Microb. Ecol..

[65] Grice E.A., Kong H.H., Renaud G., Young A.C., Program N.C.S., Bouffard G.G., Blakesley R.W., Wolfsberg T.G., Turner M.L., Segre J.A. (2008). A diversity profile of the human skin microbiota. Genome Res..

[66] Adak A., Maity C., Ghosh K., Pati B., Mondal K. (2013). Dynamics of predominant microbiota in the human gastrointestinal tract and change in luminal enzymes and immunoglobulin profile during high-altitude adaptation. Folia Microbiol..

[67] Hanski I., von Hertzen L., Fyhrquist N., Koskinen K., Torppa K., Laatikainen T., Karisola P., Auvinen P., Paulin L., Mäkelä M. (2012). Environmental biodiversity, human microbiota, and allergy are interrelated. Proc. Natl. Acad. Sci. USA.

[68] Hellmann H., Mooney S. (2010). Vitamin B6: A molecule for human health?. Molecules.

[69] Jiao S., Chen W., Wei G. (2017). Biogeography and ecological diversity patterns of rare and abundant bacteria in oil-contaminated soils. Mol. Ecol..

[70] Lynch M.D., Neufeld J.D. (2015). Ecology and exploration of the rare biosphere. Nat. Rev. Genet..

[71] Li H., Li T., Qu J. (2018). Stochastic processes govern bacterial communities from the blood of pikas and from their arthropod vectors. FEMS Microbiol. Ecol..

[72] Adair K.L., Wilson M., Bost A., Douglas A.E. (2018). Microbial community assembly in wild populations of the fruit fly. ISME J..

[73] Martinez I., Stegen J.C., Maldonado-Gomez M.X., Eren A.M., Siba P.M., Greenhill A.R., Walter J. (2015). The gut microbiota of rural papua new guineans: Composition, diversity patterns, and ecological processes. Cell Rep..

[74] Wang J., Soininen J., He J., Shen J. (2012). Phylogenetic clustering increases with elevation for microbes. Environ. Microbiol. Rep..

[75] Webb C.O., Ackerly D.D., McPeek M.A., Donoghue M.J. (2002). Phylogenies and Community Ecology. Annu. Rev. Ecol. Syst..

