# Supplementary material for: Elevation is Associated with Human Skin Microbiomes
Source: Microorganisms. 2019 Nov 24;7(12):611. doi: 10.3390/microorganisms7120611 (PMC6955857; doi:10.3390/microorganisms7120611)
Supplement: Supplementary file 1 [file microorganisms-07-00611-s001.pdf]

Supporting information

Elevation is associated with human skin microbiomes

Huan Li[1, 4]*, Yijie Wang[1], Qiaoling Yu[1], Tianshu Feng[1], Rui Zhou[1], Liye Shao[2], Jiapeng Qu[3, 4]*, Nan Li[5], Tingbei Bo[6] and Huakun Zhou[3, 4]

1. School of Public Health, Lanzhou University, Lanzhou 730000, China
2. Key Laboratory of Health Aquaculture and Product Processing in Dongting Lake Area of Hunan Province, Zoology Key Laboratory of Hunan Higher Education, Hunan University of Arts and Science, Hunan Changde 415000, China
3. Key Laboratory of Adaptation and Evolution of Plateau Biota, Northwest Institute of Plateau Biology, Chinese Academy of Sciences, Xining, Qinghai, 810008, China
4. Key laboratory of restoration ecology of cold area in Qinghai Province, Northwest Institute of Plateau Biology, Chinese Academy of Sciences, Xining, 810008, China
5. Key Laboratory of Environment Change and Resources Use in Beibu Gulf, Ministry of Education, Nanning Normal University, 175 Mingxiu East Road, Nanning, Guangxi, 530001, PR China.
6. State Key Laboratory of Integrated Management of Pest Insects and Rodents, Institute of Zoology, Chinese Academy of Sciences, Beijing 100101, China

*Corresponding authors, H.Li, E-mail: lihuan@lzu.edu.cn; J. Qu, jpqu@nwipb.cas.cn

Running title: Human skin microbiome assembly across elevations

Table S1. Sample information in this study.

Sample ID	Individual	Location	Elevation (mASL)	Weight (kg）	Height（m）	Age	Gender	UV(uW/cm^2)
P393	P31	Forehead	501	58	1.68	25	Male	53
P399	P33	Forehead	501	50	1.65	26	Male	53
P402	P34	Forehead	501	50	1.58	22	Female	53
P408	P36	Forehead	501	45	1.51	25	Female	53
P411	P37	Forehead	501	46	1.58	22	Female	53
P391	P31	Opisthenar	501	58	1.68	25	Male	53
P397	P33	Opisthenar	501	50	1.65	26	Male	53
P400	P34	Opisthenar	501	50	1.58	22	Female	53
P406	P36	Opisthenar	501	45	1.51	25	Female	53
P409	P37	Opisthenar	501	46	1.58	22	Female	53
P392	P31	Palm	501	58	1.68	25	Male	53
P398	P33	Palm	501	50	1.65	26	Male	53
P401	P34	Palm	501	50	1.58	22	Female	53
P407	P36	Palm	501	45	1.51	25	Female	53
P410	P37	Palm	501	46	1.58	22	Female	53
P378	P26	Forehead	2298	70	1.9	26	Male	1923
P381	P27	Forehead	2298	75	1.7	26	Male	1923
P387	P29	Forehead	2298	71	1.76	31	Male	1923

P390	P30	Forehead	2298	69	1.74	30	Male	1923
P417	P40	Forehead	2298	71.5	1.75	28	Male	1923
P376	P26	Opisthenar	2298	70	1.9	26	Male	1923
P379	P27	Opisthenar	2298	75	1.7	26	Male	1923
P382	P30	Opisthenar	2298	69	1.74	30	Male	1923
P385	P29	Opisthenar	2298	71	1.76	31	Male	1923
P418	P40	Opisthenar	2298	71.5	1.75	28	Male	1923
P377	P26	Palm	2298	70	1.9	26	Male	1923
P386	P29	Palm	2298	71	1.76	31	Male	1923
P389	P30	Palm	2298	69	1.74	30	Male	1923
P416	P27	Palm	2298	75	1.7	26	Male	1923
P419	P40	Palm	2298	71.5	1.75	28	Male	1923
P363	P21	Forehead	2690	55	1.65	39	Female	2318
P366	P22	Forehead	2690	48	1.65	52	Female	2318
P369	P23	Forehead	2690	45	1.65	45	Female	2318
P375	P25	Forehead	2690	74	1.78	65	Male	2318
P414	P38	Forehead	2690	55	1.6	35	Female	2318
P361	P21	Opisthenar	2690	55	1.65	39	Female	2318
P364	P22	Opisthenar	2690	48	1.65	52	Female	2318
P367	P23	Opisthenar	2690	45	1.65	45	Female	2318
P373	P25	Opisthenar	2690	74	1.78	65	Male	2318
P412	P38	Opisthenar	2690	55	1.6	35	Female	2318
P362	P21	Palm	2690	55	1.65	39	Female	2318
P365	P22	Palm	2690	48	1.65	52	Female	2318
P368	P23	Palm	2690	45	1.65	45	Female	2318
P374	P25	Palm	2690	74	1.78	65	Male	2318

P413	P38	Palm	2690	55	1.6	35	Female	2318
P303	P1	Forehead	3110	32.5	1.7	62	Male	2857
P306	P2	Forehead	3110	60	1.6	52	Female	2857
P309	P3	Forehead	3110	61.5	1.5	34	Female	2857
P312	P4	Forehead	3110	46.5	1.6	37	Female	2857
P315	P5	Forehead	3110	70	1.69	46	Male	2857
P301	P1	Opisthenar	3110	32.5	1.7	62	Male	2857
P304	P2	Opisthenar	3110	60	1.6	52	Female	2857
P307	P3	Opisthenar	3110	61.5	1.5	34	Female	2857
P310	P4	Opisthenar	3110	46.5	1.6	37	Female	2857
P313	P5	Opisthenar	3110	70	1.69	46	Male	2857
P302	P1	Palm	3110	32.5	1.7	62	Male	2857
P305	P2	Palm	3110	60	1.6	52	Female	2857
P308	P3	Palm	3110	61.5	1.5	34	Female	2857
P311	P4	Palm	3110	46.5	1.6	37	Female	2857
P314	P5	Palm	3110	70	1.69	46	Male	2857
P333	P11	Forehead	3150	60	1.68	67	Male	2943
P336	P12	Forehead	3150	70	1.7	37	Male	2943
P339	P13	Forehead	3150	65	1.65	64	Male	2943
P342	P14	Forehead	3150	65	1.6	63	Male	2943
P345	P15	Forehead	3150	60	1.65	64	Male	2943
P331	P11	Opisthenar	3150	60	1.68	67	Male	2943
P334	P12	Opisthenar	3150	70	1.7	37	Male	2943
P337	P13	Opisthenar	3150	65	1.65	64	Male	2943
P340	P14	Opisthenar	3150	65	1.6	63	Male	2943
P343	P15	Opisthenar	3150	60	1.65	64	Male	2943

P332	P11	Palm	3150	60	1.68	67	Male	2943
P335	P12	Palm	3150	70	1.7	37	Male	2943
P338	P13	Palm	3150	65	1.65	64	Male	2943
P341	P14	Palm	3150	65	1.6	63	Male	2943
P344	P15	Palm	3150	60	1.65	64	Male	2943
P348	P16	Forehead	3271	93	1.76	64	Male	3179
P351	P17	Forehead	3271	66	1.7	72	Male	3179
P354	P18	Forehead	3271	60	1.7	44	Male	3179
P357	P19	Forehead	3271	46.5	1.5	60	Female	3179
P360	P20	Forehead	3271	43.5	1.52	25	Female	3179
P346	P16	Opisthenar	3271	93	1.76	64	Male	3179
P349	P17	Opisthenar	3271	66	1.7	72	Male	3179
P352	P18	Opisthenar	3271	60	1.7	44	Male	3179
P355	P19	Opisthenar	3271	46.5	1.5	60	Female	3179
P358	P20	Opisthenar	3271	43.5	1.52	25	Female	3179
P347	P16	Palm	3271	93	1.76	64	Male	3179
P350	P17	Palm	3271	66	1.7	72	Male	3179
P353	P18	Palm	3271	60	1.7	44	Male	3179
P356	P19	Palm	3271	46.5	1.5	60	Female	3179
P359	P20	Palm	3271	43.5	1.52	25	Female	3179
P318	P6	Forehead	3431	80	1.75	47	Male	3785
P321	P7	Forehead	3431	75	1.6	37	Male	3785
P324	P8	Forehead	3431	57.5	1.7	23	Male	3785
P327	P9	Forehead	3431	62.5	1.65	51	Male	3785
P330	P10	Forehead	3431	70	1.8	40	Male	3785
P316	P6	Opisthenar	3431	80	1.75	47	Male	3785

P319	P7	Opisthenar	3431	75	1.6	37	Male	3785
P322	P8	Opisthenar	3431	57.5	1.7	23	Male	3785
P325	P9	Opisthenar	3431	62.5	1.65	51	Male	3785
P328	P10	Opisthenar	3431	70	1.8	40	Male	3785
P317	P6	Palm	3431	80	1.75	47	Male	3785
P320	P7	Palm	3431	75	1.6	37	Male	3785
P323	P8	Palm	3431	57.5	1.7	23	Male	3785
P326	P9	Palm	3431	62.5	1.65	51	Male	3785
P329	P10	Palm	3431	70	1.8	40	Male	3785

Table S2. PERMANOVA analysis showing the impacting factor of human skin microbiota functions.

	Bray-Curtis distance	
Group	*r*	*P* value
Individual	0.743	**<0.001**
Elevation	0.138	**<0.001**
Gender	0.146	**<0.001**
Age	0.102	**<0.001**
Weight	0.025	**0.039**
Height	0.021	0. 052
Body site	0.017	0. 543

Significant difference is bold in font with $P < 0.05$

Table S3. The differences of predicted gene functional abundance at level 3 between high- and low-elevation skin microbiotas.

Forehead	High_mean	Low_mean	*P*
Organismal Systems; Endocrine System; Melanogenesis	1.70E-06	**6.64E-06**	0.0000
Environmental Information Processing; Signaling Molecules and Interaction; Glycan bindng proteins	6.64E-08	**9.32E-07**	0.0000
Environmental Information Processing; Signaling Molecules and Interaction; Neuroactive ligand-receptor interaction	4.66E-07	**3.03E-06**	0.0000
Organismal Systems; Digestive System; Pancreatic secretion	5.45E-07	**3.08E-06**	0.0000
Metabolism; Metabolism of Terpenoids and Polyketides; Sesquiterpenoid biosynthesis	1.21E-06	**9.50E-06**	0.0001
Unclassified; Metabolism; Metabolism of cofactors and vitamins	1.69E-03	**1.90E-03**	0.0001
Environmental Information Processing; Signal Transduction; VEGF signaling pathway	2.39E-07	**3.38E-06**	0.0005
Metabolism; Metabolism of Terpenoids and Polyketides; Biosynthesis of 12-, 14- and 16-membered macrolides	3.83E-06	**1.46E-05**	0.0006
Metabolism; Amino Acid Metabolism; Alanine, aspartate and glutamate metabolism	**9.52E-03**	9.02E-03	0.0016
Environmental Information Processing; Signaling Molecules and Interaction; CAM ligands	8.13E-08	**4.75E-07**	0.0019
Environmental Information Processing; Signaling Molecules and Interaction; ECM-receptor interaction	8.13E-08	**4.75E-07**	0.0019
Genetic Information Processing; Replication and Repair; Homologous recombination	**7.84E-03**	7.00E-03	0.0033
Metabolism; Metabolism of Cofactors and Vitamins; Vitamin B6 metabolism	**1.92E-03**	1.79E-03	0.0044
Metabolism; Energy Metabolism; Sulfur metabolism	3.09E-03	**3.40E-03**	0.0050
Metabolism; Metabolism of Terpenoids and Polyketides; Biosynthesis of type II polyketide backbone	2.79E-06	**1.35E-05**	0.0058
Metabolism; Metabolism of Other Amino Acids; D-Arginine and D-ornithine metabolism	5.99E-05	**1.08E-04**	0.0061
Metabolism; Xenobiotics Biodegradation and Metabolism; 1,1,1-Trichloro-2,2-bis(4-chlorophenyl)ethane (DDT) degradation	1.76E-05	**3.70E-05**	0.0083
Metabolism; Biosynthesis of Other Secondary Metabolites; Betalain biosynthesis	1.33E-05	**2.79E-05**	0.0092

Opisthenar	High_mean	Low_mean	*P*
Environmental Information Processing; Signal Transduction; VEGF signaling pathway	2.54E-07	**2.72E-06**	0.0000
Metabolism; Metabolism of Terpenoids and Polyketides; Sesquiterpenoid biosynthesis	1.29E-06	**9.28E-06**	0.0000
Environmental Information Processing; Signaling Molecules and Interaction; Neuroactive ligand-receptor interaction	4.99E-07	**3.07E-06**	0.0000
Organismal Systems; Endocrine System; Melanogenesis	1.64E-06	**6.63E-06**	0.0000

Organismal Systems; Digestive System; Pancreatic secretion	5.85E-07	**3.15E-06**	0.0000
Metabolism; Metabolism of Cofactors and Vitamins; Vitamin B6 metabolism	**1.94E-03**	1.74E-03	0.0000
Metabolism; Metabolism of Terpenoids and Polyketides; Biosynthesis of type II polyketide backbone	3.26E-06	**1.26E-05**	0.0001
Metabolism; Metabolism of Terpenoids and Polyketides; Biosynthesis of 12-, 14- and 16-membered macrolides	4.07E-06	**1.44E-05**	0.0002
Environmental Information Processing; Signaling Molecules and Interaction; Glycan bindng proteins	8.86E-08	**7.28E-07**	0.0004
Environmental Information Processing; Signaling Molecules and Interaction; CAM ligands	5.35E-08	**5.26E-07**	0.0006
Environmental Information Processing; Signaling Molecules and Interaction; ECM-receptor interaction	5.35E-08	**5.26E-07**	0.0006
Metabolism; Metabolism of Cofactors and Vitamins; Nicotinate and nicotinamide metabolism	**4.56E-03**	4.12E-03	0.0009
Metabolism; Amino Acid Metabolism; Alanine, aspartate and glutamate metabolism	**9.56E-03**	8.97E-03	0.0009
Metabolism; Biosynthesis of Other Secondary Metabolites; Flavone and flavonol biosynthesis	2.03E-05	**6.11E-05**	0.0035
Unclassified; Cellular Processes and Signaling; Cell motility and secretion	2.08E-03	**2.58E-03**	0.0058
Cellular Processes; Cell Communication; Focal adhesion	4.16E-07	**1.25E-06**	0.0068
Metabolism; Biosynthesis of Other Secondary Metabolites; Betalain biosynthesis	1.32E-05	**2.90E-05**	0.0075
Genetic Information Processing; Folding, Sorting and Degradation; Protein export	**5.66E-03**	5.14E-03	0.0089

Palm	High_mean	Low_mean	*P*
Environmental Information Processing; Signal Transduction; VEGF signaling pathway	2.83E-07	**2.70E-06**	0.0000
Metabolism; Metabolism of Terpenoids and Polyketides; Sesquiterpenoid biosynthesis	1.54E-06	**9.33E-06**	0.0000
Organismal Systems; Endocrine System; Melanogenesis	2.00E-06	**6.72E-06**	0.0000
Environmental Information Processing; Signaling Molecules and Interaction; Neuroactive ligand-receptor interaction	6.01E-07	**3.16E-06**	0.0000
Organismal Systems; Digestive System; Pancreatic secretion	6.75E-07	**3.22E-06**	0.0000
Metabolism; Amino Acid Metabolism; Alanine, aspartate and glutamate metabolism	**9.54E-03**	8.99E-03	0.0007
Environmental Information Processing; Signaling Molecules and Interaction; Glycan bindng proteins	3.03E-08	**9.11E-07**	0.0008
Organismal Systems; Circulatory System; Cardiac muscle contraction	2.26E-04	**3.97E-04**	0.0008
Environmental Information Processing; Signaling Molecules and Interaction; CAM ligands	9.35E-08	**5.56E-07**	0.0042
Environmental Information Processing; Signaling Molecules and Interaction; ECM-receptor interaction	9.35E-08	**5.56E-07**	0.0042

Metabolism; Metabolism of Cofactors and Vitamins; Vitamin B6 metabolism	**1.91E-03**	1.77E-03	0.0049
Metabolism; Biosynthesis of Other Secondary Metabolites; Flavone and flavonol biosynthesis	2.47E-05	**6.36E-05**	0.0058
Cellular Processes; Cell Communication; Focal adhesion	3.45E-07	**1.14E-06**	0.0078

Only those gene functions between two groups with $P < 0.01$ were shown.Those gene functional abundance that enriched in a group was bold.

Table S4. The taxonomy and abundance of keystone species in the skin microbial community network.

Node names	Degree	Betweenesscentrality	Abundance(Mean±SE%)	Abundance ranking	Taxa
OTU151396	130	584.57	0.0250±0.0004	377th	k__Bacteria; p__Bacteroidetes; c__[Saprospirae]; o__[Saprospirales]; f__Chitinophagaceae; g__Sediminibacterium; s__
OTU9134	126	509.80	0.000060±0.000006	31962th	k__Bacteria; p__Bacteroidetes; c__Cytophagia; o__Cytophagales; f__Cytophagaceae; g__; s__
OTU178595	122	283.24	0.0490±0.0007	241th	k__Bacteria; p__Proteobacteria; c__Gammaproteobacteria; o__Xanthomonadales; f__Sinobacteraceae; g__Steroidobacter; s__
OTU162372	121	285.76	0.0362±0.0006	291th	k__Bacteria; p__Actinobacteria; c__Actinobacteria; o__Actinomycetales; f__Glycomycetaceae; g__Glycomyces; s__
OTU189624	120	294.02	0.0507±0.0008	236th	k__Bacteria; p__Bacteroidetes; c__[Saprospirae]; o__[Saprospirales]; f__Chitinophagaceae; g__Chitinophaga; s__
OTU132746	120	269.99	0.0314±0.0005	324th	k__Bacteria; p__Planctomycetes; c__Planctomycetia; o__Pirellulales; f__Pirellulaceae; g__; s__
OTU3932	117	210.81	0.0741±0.0010	160th	k__Bacteria; p__Bacteroidetes; c__Cytophagia; o__Cytophagales; f__Cytophagaceae; g__; s__
OTU59838	117	130.59	0.0239±0.0004	396th	k__Bacteria; p__Bacteroidetes; c__[Saprospirae]; o__[Saprospirales]; f__Chitinophagaceae; g__; s__
OTU11225	116	225.20	0.0218±0.0004	423th	k__Bacteria; p__Bacteroidetes; c__[Saprospirae]; o__[Saprospirales]; f__Chitinophagaceae; g__; s__
OTU154623	115	341.13	0.0200±0.0003	461th	k__Bacteria; p__Bacteroidetes; c__Cytophagia; o__Cytophagales; f__Cytophagaceae; g__; s__
OTU90794	115	287.85	0.1715±0.0030	76th	k__Bacteria; p__Actinobacteria; c__Actinobacteria; o__Actinomycetales; f__Streptosporangiaceae
OTU62896	114	189.37	0.0529±0.0007	226th	k__Bacteria; p__Verrucomicrobia; c__Opitutae; o__Opitutales; f__Opitutaceae; g__Opitutus; s__
OTU55884	113	690.49	0.0702±0.0009	169th	k__Bacteria; p__Acidobacteria; c__Solibacteres; o__Solibacterales; f__; g__; s__
OTU87324	113	200.05	0.0271±0.0004	357th	k__Bacteria; p__Actinobacteria; c__Actinobacteria; o__Actinomycetales; f__Micromonosporaceae; g__Catellatospora; s__
OTU183848	112	175.57	0.1151±0.0017	104th	k__Bacteria; p__Bacteroidetes; c__[Saprospirae]; o__[Saprospirales]; f__Chitinophagaceae; g__Flavisolibacter; s__
OTU64249	110	184.81	0.1171±0.0016	99th	k__Bacteria; p__Bacteroidetes; c__[Saprospirae]; o__[Saprospirales]; f__Chitinophagaceae; g__Flavisolibacter; s__
OTU15771	110	111.25	0.0250±0.0004	379th	k__Bacteria; p__Bacteroidetes; c__[Saprospirae]; o__[Saprospirales]; f__Chitinophagaceae; g__Flavisolibacter; s__
OTU113663	110	101.58	0.0711±0.0010	168th	k__Bacteria; p__Bacteroidetes; c__Cytophagia; o__Cytophagales; f__Cytophagaceae; g__; s__
OTU173264	109	131.14	0.0380±0.0006	283th	k__Bacteria; p__Bacteroidetes; c__[Saprospirae]; o__[Saprospirales]; f__Chitinophagaceae
OTU40259	108	169.40	0.0197±0.0003	473th	k__Bacteria; p__Bacteroidetes; c__Cytophagia; o__Cytophagales; f__Cytophagaceae; g__; s__
OTU54118	108	90.47	0.0165±0.0003	539th	k__Bacteria; p__Proteobacteria; c__Alphaproteobacteria; o__Sphingomonadales; f__Sphingomonadaceae; g__Kaistobacter; s__
OTU55772	108	75.94	0.0216±0.0003	428th	k__Bacteria; p__Bacteroidetes; c__Cytophagia; o__Cytophagales; f__Cytophagaceae; g__; s__
OTU95921	107	966.84	0.1767±0.0022	71th	k__Bacteria; p__Acidobacteria; c__[Chloracidobacteria]; o__RB41; f__; g__; s__
OTU38048	106	173.91	0.0282±0.0004	346th	k__Bacteria; p__Bacteroidetes; c__[Saprospirae]; o__[Saprospirales]; f__Chitinophagaceae; g__; s__

OTU183622	104	46.55	0.1343±0.0020	89th	k__Bacteria; p__Bacteroidetes; c__Cytophagia; o__Cytophagales; f__Cytophagaceae; g__; s__
OTU18516	103	99.87	0.1418±0.0022	84th	k__Bacteria; p__Proteobacteria; c__Gammaproteobacteria; o__Xanthomonadales; f__Sinobacteraceae; g__Steroidobacter; s__
OTU42013	103	93.55	0.0555±0.0008	215th	k__Bacteria; p__Proteobacteria; c__Betaproteobacteria; o__SC-I-84; f__; g__; s__
OTU177470	101	1463.77	0.2595±0.0032	55th	k__Bacteria; p__Bacteroidetes; c__[Saprospirae]; o__[Saprospirales]; f__Chitinophagaceae; g__Flavisolibacter; s__
OTU31632	101	97.68	0.0134±0.0002	625th	k__Bacteria; p__Proteobacteria; c__Deltaproteobacteria; o__Myxococcales; f__; g__; s__
OTU181707	101	66.33	0.0338±0.0005	306th	k__Bacteria; p__Proteobacteria; c__Alphaproteobacteria; o__Sphingomonadales; f__Sphingomonadaceae; g__Kaistobacter; s__
OTU106567	101	57.97	0.0245±0.0004	386th	k__Bacteria; p__Proteobacteria; c__Deltaproteobacteria; o__Myxococcales; f__Polyangiaceae; g__Sorangium; s__cellulosum

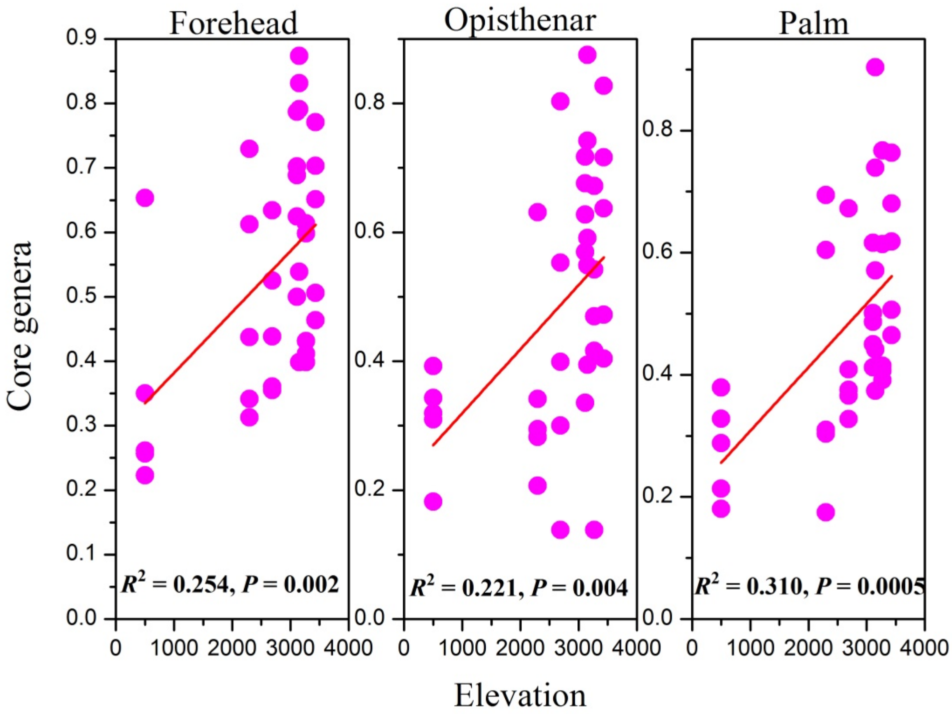

Figure S1. The linear regression relationship between elevation and the total abundance of core genera in different body sites

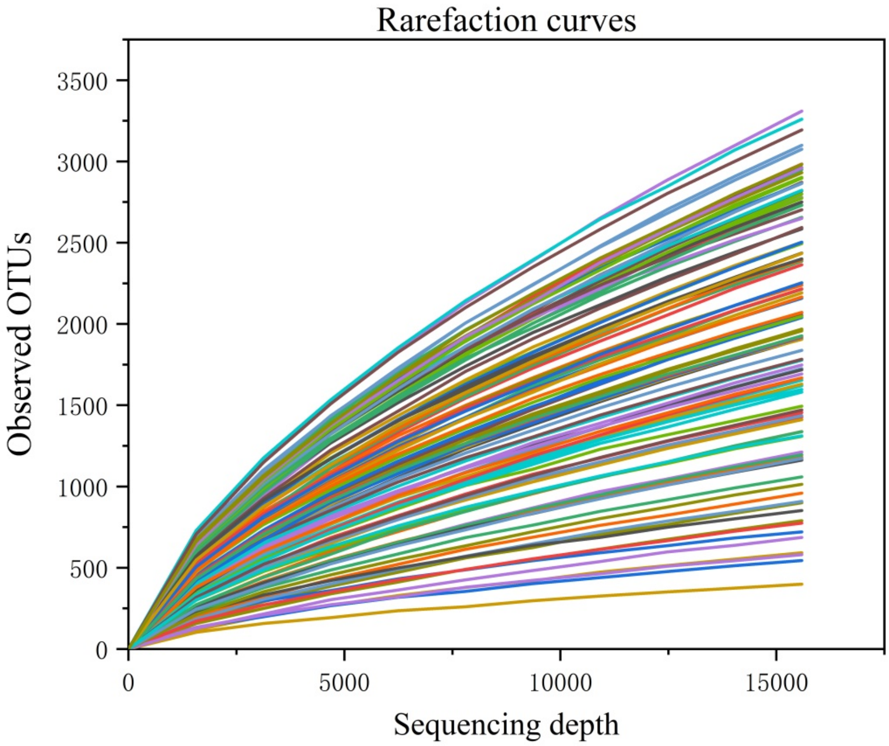

Figure S2. The rarefaction curves of observed OTUs at OTU level across all samples.

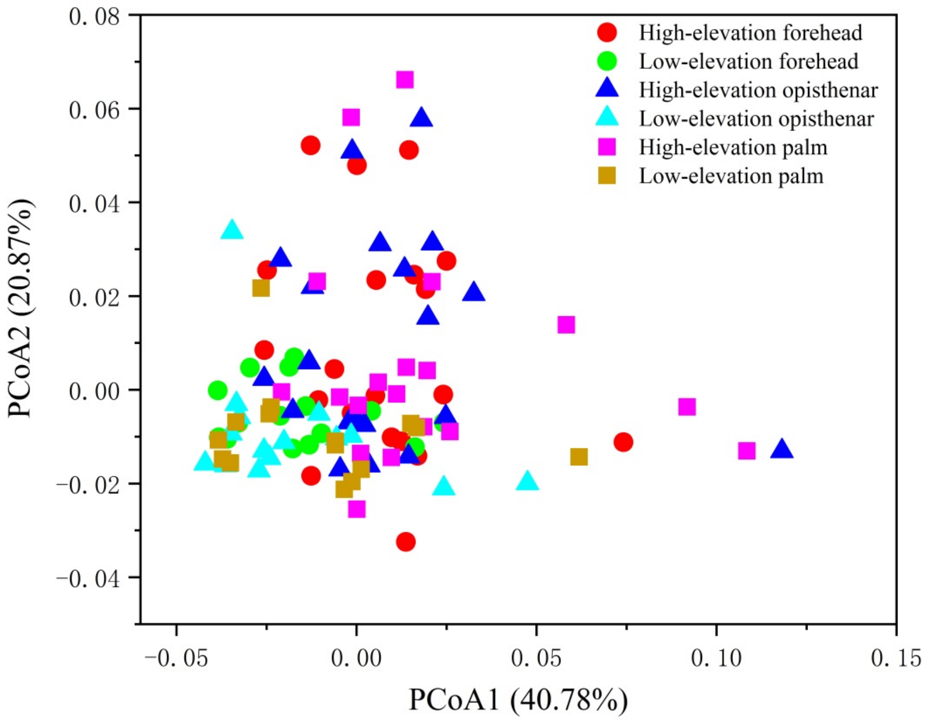

Figure S3. Principal coordinate analysis (PCoA) showing the difference of functional structure between high- and low-elevation skin microbiota based on predicted metagenomics at level 3 of KEGG.
